# Social care planning and provision for people with young onset dementia and their families: Protocol for the DYNAMIC study

**DOI:** 10.1371/journal.pone.0297747

**Published:** 2024-02-05

**Authors:** Catherine Quinn, Helen Young, Kate Gridley, Vasileios Stamou, Clare Mason, Jan Oyebode

**Affiliations:** 1 Centre for Applied Dementia Studies, University of Bradford, Bradford, United Kingdom; 2 Wolfson Centre for Applied Health Research, Bradford, United Kingdom; 3 Social Policy Research Unit, University of York, York, United Kingdom; PLoS ONE, UNITED STATES

## Abstract

**Background:**

Social care is vital to quality of life for people with young onset dementia and their families. Yet care is hugely variable, frequently lacking and poorly coordinated. We aim to establish current practice in English social care for people with young onset dementia and co-produce evidence-based recommendations and resources for improvement.

**Methods and analysis:**

In Work-Package 1, we will gather qualitative data from 25 people with young onset dementia and/or main supporters residing in England. We will ask them about their experiences of social care (broadly defined, including independent and voluntary sector provision) and suggestions for improvement. In Work-Package 2, we will conduct a short on-line survey with a wide range of staff with a role in adult social care in England. We will find out about current awareness, knowledge and practice and suggestions for improvements. Quantitative and qualitative analysis will provide a picture of current practice. In Work-Package 3, we will use convergence analysis to synthesise the findings from Work-Packages 1 and 2 and present the findings to a stakeholder workshop, to identify feasible priorities for improvement. We will establish what is already known about good practice relating to these key priorities using a scoping review and interviews with professionals. This knowledge will then feed into the co-production of resources and recommendations with key stakeholders to improve social care for people with young onset dementia and their families.

**Discussion:**

This study seeks to address a gap in our understanding of social care provision for people with young onset dementia and develop recommendations and practical resources for improvements. The findings will help people with young onset dementia and supporters to receive higher quality social care.

**Trial registration:**

**Study registration number:** ISRCTN10653250.

## Introduction

It is estimated that 55 million people are currently living with dementia worldwide, a number estimated to increase to 139 million by 2050 [1]. There are many different types of dementia. Although young onset dementia (dementia with an onset under 65 years of age) is relatively rare, its prevalence in the UK at 70,800 [2] is higher than other well-known conditions, e.g. over 8 times as many people have young onset dementia than have motor neuron disease [3]. The condition has great impact on those diagnosed and costs families the equivalent of £10,000 in family care per 3 months [4]. There is also a wider cost to society as both the person diagnosed and/or their main supporter may be unable to work [5]. There are also long-term consequences for the well-being, education and mental health of children/young people in the family [6, 7]. Formal services currently fund little care or support for people with young onset dementia, unless the person is admitted to residential care [4].

Social care has an important role in supporting individuals. The purpose of social care planning is to enable people to remain independent, have control over their life, do things they enjoy, know what type of care may help and understand their condition and care needs [8, 9]. Social care is vital to the quality of life of people with young onset dementia and their families [10, 11] but their social care needs are seldom well addressed [4, 6, 12]. Research in the Angela Project found that almost a third of people living with young onset dementia had no regular appointments with any social or health care professional and fewer than 1 in 3 had a care plan [6]. Lack of social care has far-reaching consequences, exposing people with young onset dementia and their families to risks, including strained relationships, financial hardship, mental distress and ill-being. Unmet social care needs are significantly associated with distress and psychiatric symptoms [13]. They may precipitate crises for the person with young onset dementia and their families [7]. Services may then have to step in to address issues that could have been averted by earlier social care provision. There is a great need to improve social care for people with young onset dementia to address the inequalities experienced by this population.

There is little published research and no published reviews specifically addressing young onset dementia social care. A 2016 review on young onset dementia [12] found only 10 sources focused on service experiences, concluding that there was: ‘lack of a clear diagnostic pathway, poor provision of information, lack of appropriate referrals to support services, and a high volume of informal care provided’ (p.5). A 2018 systematic review [10] of service provision for people with young onset dementia located six UK-based studies on social care needs and service provision. The authors concluded these initiatives enabled people with young onset dementia to continue living at home for longer but also that: ‘The evidence on the experience of living with young onset dementia is not matched by research and the innovation needed to mitigate the impact of young onset dementia’ (p.933). A 2022 meta-synthesis [8] identified 11 publications on views of specific supportive services and found there was great overlap in preferences of those with young onset dementia and their carers. The authors identified the need for further studies that include both people with young onset dementia and their carers and focus specifically on services. Against this backdrop of limited research, the Angela project [14], identified eight key needs of people with young onset dementia and family members, highlighting a strong social care component [15, 16]. The project confirmed that care was hugely variable, frequently lacking and poorly coordinated [4]. In particular, the person with young onset dementia was often discharged from secondary to primary health care, with little focus on social care [4]. Due to the predominantly healthcare focus of the Angela project, findings on social care were patchy and ‘fragmented’. Additionally, as the Angela project focused on positive experiences of diagnostic and post-diagnostic services, it did not identify unmet social care needs or shortcomings in services. Given the lack of research in this area, it is essential to establish gaps in social care practice and areas for improvement to inform the development of recommendations and resources to improve social care for people with young onset dementia.

Research [4, 10–12, 15, 17] has established the high level and breadth of social care needs in young onset dementia, the lack of social care for young onset dementia and the deleterious consequences of living with unmet social care needs for the person [13], main supporter [4, 17] and children/young people within the family [6, 7]. When developing this study our consultation work with a range of stakeholders indicated that no-one seems to have a clear picture of current social care for young onset dementia. In this project, we aim to address this gap and co-produce evidence-based recommendations.

## Objectives

The DYNAMIC study aims to:

1. Gain in-depth understanding of the social care needs, experiences and preferences of those living with young onset dementia and their supporters.
2. Establish levels of awareness, knowledge and practice among professionals regarding social care needs, care planning, and provision for people with young onset dementia.
3. Co-produce recommendations and resources to improve social care for people with young onset dementia and their supporters.

## Materials and methods

In this study we aim to establish current practice in English social care for people with young onset dementia and co-produce evidence-based recommendations and resources to improve social care. This study involves two stages: The first stage (to establish current practice) incorporates Work-packages 1 and 2 and Work-package 3 (to co-produce evidence-based recommendations and resources) will comprise the second stage.

### Work-package 1

In this Work-package we will employ qualitative methods to better understand experiences of social care and areas for improvement from the perspectives of people living with young onset dementia and their main supporters. Recruitment for this Work-package commenced on 3/11/2023.

We will recruit 25 ‘cases’, with each case constituting an individual with young onset dementia and/or a main supporter (typically a family member or friend), from England. People with young onset dementia who do not have a main supporter will still be eligible to participate in the study. The inclusion and exclusion criteria are provided in Box 1.

**Box 1**

**INCLUSION CRITERIA**

For people with dementia:

1. Diagnosed with young onset dementia (defined as when the first symptoms of dementia occur before the age of 65 years)
2. Living alone or living with others

For main supporters:

1. Relatives, friends or neighbours self-identified as involved in supporting a person with young onset dementia.
2. May be living within or outside the home environment of the person with dementia.

Staff will be:

1. Staff with a role in or awareness of adult/older adult social care planning, provision, management or commissioning

**EXCLUSION CRITERIA**

People with dementia will be excluded if they are any of the following:

1. Diagnosed with dementia caused by HIV, traumatic brain injury, Down’s syndrome, Huntington’s chorea or alcohol-related dementia.
2. Lack capacity to consent.
3. Unable to communicate.
4. Residing outside of England.

Main supporters will be excluded if they are any of the following:

1. Not caring for someone with young onset dementia
2. Caring for the person in a professional capacity e.g. paid carers.
3. Residing outside of England

Staff will be excluded if they:

1. Do not have a role in or awareness of adult/older adult social care planning, provision, management or commissioning.
2. Work outside of England.

We will raise awareness of the study and recruit potential participants through a variety of media, including face-to-face meetings, with the help of national and local charitable and community-based organisations. Purposive sampling will ensure variation in characteristics that may influence experiences of social care e.g. gender, ethnicity and type of dementia. We will ensure inclusion and representation through consultation with patient and public involvement (PPI) members of the project team about the recruitment strategies. People who express an interest in taking part will be screened to ensure they meet the inclusion criteria.

We will offer a range of ways for people to express interest in taking part including in person (when the researcher visits community and peer support groups), online (by clicking a link or using a QR code), or by directly phoning or emailing the researcher. The researcher will contact those who express an interest to answer any questions about the study and check the individual meets the inclusion criteria. If the person meets these criteria the researcher will send them the information sheet (through email or post) and follow this up with further phone/email contact. Those who are ineligible or do not wish to participate will have their details destroyed. If the person has capacity to give informed consent and decides to take part the researcher will arrange a date and time for the research interview. The interview will take place in person, online or over the phone depending on the preference of the individual. Those with young onset dementia will be given a choice of having someone they trust present to support them. If both a person with young onset dementia and a supporter take part, they will be given a choice to be interviewed jointly or individually. Participants will be contacted with a reminder about the interview through their preferred contact method 1–2 days before the date of the meeting. In-person interviews will be conducted at the home of the interviewee or a mutually agreed suitable location, e.g. University premises.

On the day of the interview the researcher will re-confirm the participant’s capacity to consent and obtain consent either orally or in writing before the interview commences. Interviews will take the form of an extended conversation [18] guided by a semi-structured topic guide, allowing participants to focus on issues of most importance to them and enabling the researcher to probe for greater depth. Potential topics include experiences of social care; social care needs (met and unmet); contextual factors and nuances of the person’s unique situation, and suggested improvements or changes to social care provision. The interviews will last approximately 30–45 minutes but could be conducted over two occasions at participants’ requests. Interviews will be digitally recorded and transcribed. Participants will be given a voucher as a token of appreciation for their time.

Data will be managed using the Framework approach [19] and analysed inductively using reflexive thematic analysis [20]. We will take an ‘experiential’ approach, focusing on the meanings and experiences articulated by participants, underpinned by hermeneutics of empathy [20]. From this approach, themes are ‘conceptually founded patterns’ [21] reflexively developed by the research team but grounded in the expressed experiences of participants. After initial familiarisation and summarising, development and refinement of themes will be undertaken using a series of case by theme matrices (frameworks) to enable us to visualise relationships between cases, topics and themes. Involvement of the project team (including PPI members) at key points will bring in multiple perspectives, reducing the influence of any one researcher and ensuring rigour and accountability throughout [22].

### Work-package 2

In this work-package we will employ a quantitative methodology to establish awareness, knowledge and practice among staff regarding social care needs, care planning and provision for people with young onset dementia. This will involve a short on-line survey distributed across England.

Participants will be staff with a role in or awareness of adult social care planning, provision, management or commissioning. Staff could be employed by Local Authorities, the third sector, the healthcare sector, the private sector or integrated care boards. They do not have to have seen people with young onset dementia but need to have a remit that could include people with young onset dementia. The inclusion and exclusion criteria are listed in Box 1. To ensure recruitment across the whole range of social care professionals, we aim to establish the size and nature of the key population/ subpopulations (e.g., social workers, home carers, social prescribers) and check the distribution of respondents two and four months into recruitment. This will allow us to target later recruitment drives to increase representation from under-represented groups. Socio-demographic questions will enable us to check diversity of recruitment and this will allow focused recruitment campaigns.

The survey will be publicised through social media, key contacts of the research team and existing networks such as the Association of Directors of Adult Social Services. The publicity leaflet will include a link to the online survey. Contact details of the researcher will be provided so that those interested in taking part can find out more information about the study. The information sheet will be accessible through a link in the online survey or posted to those participants who request it. The survey will be available on paper for participants who do not have access to the internet or prefer to complete a paper version.

The survey will be kept concise and primarily consist of closed-ended questions, with some open-ended questions to enable participants to elaborate on their responses. Survey items will be informed by previous research and consultation with PPI members of the project team. Questions will address frequency of contact with people with young onset dementia; practice in the provision of social care in young onset dementia; and perceived areas for improvement in social care. We will also collect participants’ socio-demographic information and geographical location (e.g. rurality and ethnicity). To ensure the appropriateness of the length, wording, and content we will pilot the survey and will also use cognitive interviewing, where the person ‘thinks aloud’, explaining their thoughts as they answer each question [23], with at least two individuals.

### Data analysis

All data will be imported into SPSS for analysis. We will produce descriptive data; for example, about the frequency of contact with people with young onset dementia. Some open-ended data will be imported into NVivo (qualitative data management software) or Excel for analysis. Content analysis [24] will be used to analyse responses to open-ended questions, and identify common themes, or categories, in participants’ responses; for example, in relation to improvements in social care. Emergent themes will be discussed with the wider research team, including PPI members, to ensure rigour in data analysis and relevance of the findings [22].

### Work-package 3

This Work-package will have several stages. The first stage comprises a synthesis of Work-package 1 and Work-package 2 findings to identify potential areas for improvement in social care provision. Themes from work-packages 1 and 2 will be compared to identify areas of convergence (similarities) and divergence (differences) [25]; e.g., if few Work-package 1 participants report receiving a social care assessment, this can be compared with Work-package 2 findings on staff reports of conducting social care assessments for people with young onset dementia. A consistent finding (e.g. few received and few delivered) could indicate a gap in provision, whereas an inconsistent finding (e.g. few received but many delivered) could indicate a mismatch of perceptions, implying a need for clearer communications. Suggestions for service change and improvement will be directly compared. These could include aspects such as improving social workers’ awareness of the impact of young onset dementia or development of more meaningful personalised care plans. The synthesised findings of Work packages 1 and 2 will be summarised for the next stage.

The second stage will involve identifying impactful feasible priorities for improvement in social care provision. Priorities will be established through an on-line consensus workshop. A purposive sample of up to 20 diverse stakeholders will be recruited through our existing contacts with organisations and individuals and the suggestions of Steering Group members. People who took part in Work-packages 1 and 2 will also be approached to take part. Stakeholders will include those with young onset dementia and main supporters, local authority and third sector staff. PPI members of the research team and the PPI lead will advise on ways to ensure meaningful involvement.

The online workshop will last approximately 90 minutes. It will begin with a presentation and discussion of the Stage One findings, followed by break-out groups focused on the broad areas of service improvement identified through Work-packages 1 and 2. To address existing issues of inequalities in access to services, one group will focus specifically on diversity. Groups will be tasked with considering the findings relevant to their service improvement area and with suggesting improvements (e.g., tools, guidelines, training interventions) that would be impactful and feasible to introduce. Each group will be facilitated by a member of the research team who will ensure the voices of people living with young onset dementia are included and represented in the process. The workshop will end with feedback from each group and the collation of a set of feasible priorities for improvement in social care [25]. The workshop notes will be typed up and each group’s comments, priorities and feasibility ratings will be checked against the collated list of feasible priorities from the day. This will result in three areas of focus to be taken forward in the remainder of the work package. A summary of the workshop will be sent to all attendees by email or other preferred means of communication. All will be invited to join a reference group to continue their involvement as the recommendations and resources are developed.

The third stage will comprise a scoping review and interviews with professionals to establish existing knowledge on good practice within the priority areas, which will inform the development of the recommendations and resources on social care provision. A scoping review will be conducted on the key identified priorities for improvement, to explore, map and summarise relevant evidence and inform initial practice recommendations. Studies of all methodologies will be included, including grey literature. Systematic searches will be conducted on relevant databases (e.g., PubMed, Scopus, EBSCO), supplemented by reference-checking and hand-searching of relevant journals. Titles and abstracts retrieved will be reviewed against pre-defined inclusion and exclusion criteria. Full manuscripts of retained articles will then be checked against the criteria. The review process will align with the Preferred Reporting Items for Systematic Reviews and Meta-Analyses (PRISMA) Extension for Scoping reviews [26, 27]. We will appraise the quality of studies using the Mixed Methods Appraisal Tool [28] and grey literature using the AACODS (Authority, Accuracy, Coverage, Objectivity, Date, Significance) checklist [29].

Findings from the included studies will be extracted according to their relevance to the identified improvement priorities. Data analysis will involve an initial description of the sample of studies and their quality. The findings will be thematically grouped and presented descriptively. A diagrammatic map of the detailed findings will be developed, encompassing the key areas of improvement and themes/sub-themes related to key aspects of good practice for each. This will serve as a template for the analysis of the subsequent interviews with professionals.

Interviews with professionals will complement the scoping review and gather good practice examples of social care, supplemented, where possible, by anonymised copies of relevant documents (e.g. policy documents, care plan templates, examples of leaflets). The participants will be 10–12 social care practitioners/policy-makers who will be interviewed to provide data of sufficient depth and breadth [30]. We will use purposive sampling to recruit a diverse sample across sectors, with the focus depending on the particular improvements identified. Interviewees will be identified from survey respondents from Work-package 2 who agreed to be contacted, known centres of good practice, recommendations from the Project Steering group, other networks and social media. All participants will be asked to give informed consent and will also be invited to express interest in continuing involvement in the project, through joining a reference group to develop the resources and recommendations for social care improvement.

A flexible semi-structured interview guide will be developed, with advice and feedback from the PPI members of the research team and the Steering Group. It will be informed by the scoping reviews and focus on the key priorities for improvement in social care. In-depth insights and understanding of relevant examples of good practice will be sought, including participants’ understanding of the key improvement areas and what good practice in these entails (e.g. organisational and contextual factors facilitating the process, delivery format, time, budget). We will also gather suggestions for further improvement and elaborate on key facilitators and barriers. Interviews will be digitally recorded and transcribed for analysis. Data will be analysed using template analysis [31]. The diagrammatic map resulting from the scoping reviews will provide the initial template. Relevant text excerpts will be labelled according to the template, clustering new codes into pre-existing themes and/or constructing new themes from participants’ accounts. Evolving themes and codes will be discussed within the research team to bring in multiple perspectives to ensure the rigour and relevance of the findings [22].

Stage four will involve the co-production of resources and recommendations for social care improvement. We will co-produce, with stakeholders, evidence- and practice-based recommendations and resources focused on the three priorities for improvement identified by stakeholders at the consensus workshop [32]. We will follow the principles of good practice in co-production [33] including relationship-building, flexibility in arrangements, agreeing clear ground rules, inclusion and reflection. We will purposively recruit 4–6 diverse stakeholders with a particular interest in each of the three improvement priorities to act as reference groups and inform resource development. In line with co-production principles [33], the process will be tailored to the wishes of the reference groups. A short on-line meeting/email discussion with each reference group will take place at the outset to discuss and agree this. The general process will be one of iteration, with feedback cycles involving direction from reference group members and production by the researchers [34]. The templates summarising good practice will form the basis for development of tangible resources/recommendations. Initially, the reference group will identify the form of the preferred resource/ recommendation (e.g. on-line guidance, downloadable templates, videos). Once the nature of the resource is agreed a draft will be created. The reference group will then feedback on the draft. If required, this cycle will be repeated until we have generated practice- and evidence-informed recommendations and resources. The academic research team will be joined by a member of staff from DementiaUK, a national charity with a specialist arm focused on young onset dementia, who will bring expertise in the co-production of online, paper, video and other resources for people with young onset dementia, families, and health and social care professionals.

## Patient and public involvement

In developing the proposal, we consulted with people directly affected by young onset dementia and providers from social services, voluntary and health sectors. We also worked directly with two people with dementia and two main supporters. Within the project we have a dedicated PPI lead (CM), two PPI members on the Project Management Group and two PPI members on the Project Steering Group. PPI members will be involved in the development of recruitment and data collection materials, information sheets and discussions about the research findings. They will support knowledge exchange, dissemination and impact throughout the project. Additional PPI members will support the co-production work in Work-package 3. They will contribute to the development of project recommendations/ resources, through participation in: a) the stakeholder workshop which will set the priorities for improvement and b) the smaller reference groups which will co-produce the resources/recommendations and associated impact plans.

## Data management

All study documentation and data will be stored on a secure University of Bradford shared drive, accessible only to the research team. All interviews will be audio recorded on a password protected device. Recordings will be uploaded to a shared drive which will only be accessible through password protected computers. Following the uploading, the original recording will be deleted from the recording device. The audio files will be transcribed verbatim into a Word document by the researchers or by a University of Bradford approved supplier. Recordings and anonymised transcripts will be stored using a unique identification number for each participant and will be stored separately to participants’ personal details. All quotations used in the study final report, publications and presentations will be pseudonymised to ensure that no identifying details are present (e.g. names of individuals).

All Work-package 2 survey data will be entered into a secure online database. Each participant will be assigned a unique identity code. The data will be anonymised with the exception of the names of services and their location.

Participants may decide to provide personal contact details to allow the research team to make contact about following stages of the study, to hear about the findings of the study or to be informed of future studies. This data will be stored in a separate password-protected electronic file in the secure servers of the University of Bradford which will only be accessible by the research team through password protected computers.

## Discussion

The DYNAMIC study seeks to address an important topic area around social care provision for people with young onset dementia. There are distinctive social care needs associated with young onset dementia; yet due to the rarity of the condition, these needs are not well known to social care practitioners and are rarely met, leading to poorer quality of life and crises in families affected by young onset dementia. In exploring current provision, we seek to develop recommendations and resources to improve social care and support. This may result in improved quality of social care services and may have a positive impact on the quality of life and well-being of people with young onset dementia and their supporters.

A strength of this study lies in the active involvement of people with young onset dementia and their supporters in major aspects of project development and implementation. Members of our PPI group have been involved in the design of the study to ensure it is inclusive of those affected by young onset dementia. They will also be involved in different aspects of study delivery to ensure the relevance of the study and the associated findings. The study will also incorporate multiple perspectives of people with young onset dementia, supporters, and professionals, to better understand current practice in social care provision and how it can be improved. Due to the small scale of the study, we will only include people with young onset dementia who have capacity to consent. However, main supporters of people with advanced dementia will be able to participate, to ensure wide representation. The study recommendations will also be co-produced in collaboration with people with young onset dementia, supporters, and professionals, to increase the relevance and potential applicability of the findings in real-life practice.

The young-onset-dementia-specific recommendations and resources will provide tools which may improve real-life social care provision. The resources/recommendations will raise awareness of younger people’s needs among social care professionals and policy makers and may inform the re-designing of services or social care improvements beyond the few existing pockets of good practice. Our wide dissemination strategy will increase the impact of the study and may also enhance initiatives of service recipients to advocate for higher quality social care in young onset dementia.
